# Allicin Attenuated Advanced Oxidation Protein Product-Induced Oxidative Stress and Mitochondrial Apoptosis in Human Nucleus Pulposus Cells

**DOI:** 10.1155/2020/6685043

**Published:** 2020-12-14

**Authors:** Qian Xiang, Zhangrong Cheng, Juntan Wang, Xiaobo Feng, Wenbin Hua, Rongjin Luo, Bingjin Wang, Zhiwei Liao, Liang Ma, Gaocai Li, Saideng Lu, Kun Wang, Yu Song, Shuai Li, Xinghuo Wu, Cao Yang, Yukun Zhang

**Affiliations:** Department of Orthopaedics, Union Hospital, Tongji Medical College, Huazhong University of Science and Technology, Wuhan 430022, China

## Abstract

Intervertebral disc degeneration (IDD) is one of the most common chronic degenerative musculoskeletal disorders. Oxidative stress-induced apoptosis of the nucleus pulposus (NP) cells plays a key role during IDD progression. Advanced oxidation protein products (AOPP), novel biomarkers of oxidative stress, have been reported to function in various diseases due to their potential for disrupting the redox balance. The current study is aimed at investigating the function of AOPP in the oxidative stress-induced apoptosis of human NP cells and the alleviative effects of allicin during this process which was known for its antioxidant properties. AOPP were demonstrated to hamper the viability and proliferation of NP cells in a time- and concentration-dependent manner and cause cell apoptosis markedly. High levels of reactive oxygen species (ROS) and lipid peroxidation product malondialdehyde (MDA) were detected in NP cells after AOPP stimulation, which resulted in depolarized mitochondrial transmembrane potential (MTP). Correspondingly, higher levels of AOPP were discovered in the human degenerative intervertebral discs (IVD). It was also found that allicin could protect NP cells against AOPP-mediated oxidative stress and mitochondrial dysfunction via suppressing the p38-MAPK pathway. These results disclosed a significant role of AOPP in the oxidative stress-induced apoptosis of NP cells, which could be involved in the primary pathogenesis of IDD. It was also revealed that allicin could be a promising therapeutic approach against AOPP-mediated oxidative stress during IDD progression.

## 1. Introduction

Low back pain (LBP) has become an increasingly common disorder in contemporary times, lowering patients' quality of life and burthening cosmopolitan healthcare system and society economically [1, 2]. One of the leading causes of LBP is intervertebral disc degeneration (IDD) [3]. The intervertebral disc, the largest avascular organ in the human body, comprises a centric nucleus pulposus (NP) and an ambient annulus fibrosus [4]. Many previous studies demonstrate that the main pathological changes in degenerated discs include excess apoptosis of NP cells and degradation of the extracellular matrix (ECM) [5, 6]. Apoptosis is a highly conserved cellular function to remove excrescent and volatile cells in different pathophysiological processes, and plays a vital role in sustaining healthy function of tissues and organs; however, superfluous apoptosis of NP cells is the primary cause of IDD [7–9]. Recent studies have shown that excess apoptosis of NP cells is largely promoted by an imbalance of the cellular redox system in the disc microenvironment, while interventions targeting oxidative stress markedly protect NP cells from apoptosis [10, 11].

Once cellular redox homeostasis is disrupted, proteins in this environment are prone to be damaged under oxidative stress [12–14]. Advanced oxidation protein products (AOPP), which are important derivatives of such damaged proteins, mainly originate from oxidatively modified albumin and have been identified recently as circulating plasma biomarkers of oxidation stress [15]. Indeed, AOPP accumulate in the serum of patients with various chronic diseases, such as inflammatory bowel disease, diabetes, and chronic kidney disease [16]. A previous study revealed that reactive oxygen species (ROS), which are potent oxidants, can be generated by AOPP through a NADPH oxidase-dependent pathway [17]. Research on diabetic nephropathy also showed that the accumulation of AOPP can induce oxidative stress and mitochondrial injury [18]. Other studies demonstrated that AOPP can act as potent inducers of apoptosis [19, 20]. Recently, abnormal accumulation of AOPP was found in the articular cartilage of rodent osteoarthritis (OA) models and had an endogenous pathogenic role in OA progression [21]. In addition, a prior study showed that AOPP participated in age-related changes in rat lumbar IVDs [22]. Considering that IVD cells and chondrocytes share phenotypic and morphological similarities, it is important to investigate how AOPP work in IDD progression, which remains unclear thus far [23].

Many constituents of medicinal herbs, including allicin, have various benefits [24]. Allicin, one of the organosulfur compounds extracted from the garlic bulb, shows potent effects on eliminating free radicals, abating oxidative stress, and protecting mitochondrial functions [25–28]. Recent studies have also demonstrated that allicin exerts extensive therapeutic effects in various diseases, such as cardiovascular and chronic kidney disease, because of its antioxidant properties, while its influence on IDD is still unknown [28–30].

Based on the above findings, we hypothesized that the accumulation of AOPP in the disc microenvironment might contribute to oxidative stress in NP cells, which further erode mitochondrial functions and promote apoptosis. The present study was undertaken to explore the role of AOPP in oxidative stress and apoptosis of human NP cells and to determine the effect of allicin on these AOPP-mediated processes. We exposed NP cells in vitro to different levels of AOPP or allicin and gathered experimental data on multiple aspects, such as cell proliferation, apoptosis, oxidative stress, and changes in the mitochondrial membrane. In addition, as an important avenue for cell demise, the activation of MAPK pathways was reported to be crucially involved in ROS-triggered apoptosis [31, 32]. Considering the role of ROS as a pathogenic intermediary of AOPP-initiated diseases, thus, we detected the expression of key proteins in MAPK signaling pathways potentially mediating the effects of AOPP on NP cells [17]. We aimed to provide a general overview of the molecular mechanisms by which AOPP affected human NP cells and inform possible therapeutic strategies. Our research is aimed at helping to better understand the pathogenesis of IDD and providing a promising therapeutic approach to retard its progression.

## 2. Material and Methods

### 2.1. Collection of NP Tissue and Ethics Statement

To separate NP cells, the healthy NP specimens (Pfirrmann I or II) of human IVD were obtained from deformity correction surgery for 6 patients (3 females and 3 males; average age: 18.3 years; age range: 16-20 years) with idiopathic scoliosis. To compare AOPP levels, the degenerative NP tissues (Pfirrmann IV or V) were acquired from spinal decompression surgery for 3 patients (2 females and 1 male; average age 63.4 years; age range: 63-65 years) with spinal stenosis, and the healthy tissues were collected from deformity correction surgery for 3 patients (2 males and 1 female; average age 25 years; age range: 24-26 years) with idiopathic scoliosis. In brief, after being excised intraoperatively, the healthy tissues used for separating cells were instantly put into a chilled vessel with Hank's balanced salt solution, and the other healthy or degenerative tissues used for AOPP testing were snap-frozen. All samples were then transported to the laboratory for the further handling according to their intended use or cryopreserved in liquid nitrogen. Each donor was informed of the experimental usage of the NP tissue, and their approvals were acquired. Our research protocol was approved by the Clinical Research Ethics Committee of Tongji Medical College, Huazhong University of Science and Technology. All methods were applied in strict adherence with the approved guidelines.

### 2.2. Separation and Culture of NP Cells

Briefly, the fresh tissue was chopped into small pieces (1 mm^3^ or so) and digested at 37°C using 0.25% trypsin (Gibco, UK) for 30 min before 0.2% collagenase-type 2 (Gibco, UK) for 4 h in a humid environment with 5% CO_2_. The suspension was centrifuged after the pieces were filtered and washed using phosphate buffered saline (PBS). Next, the cultivation of separate cells was conducted in Dulbecco's modified Eagle' medium (DMEM) comprising nutrient admixture F12 (Gibco, USA) and 15% fetal bovine serum (FBS; Gibco, USA) and streptomycin (100 *μ*g/ml; Gibco, USA) as well as penicillin (100 units/ml; Gibco, USA). The culture medium was renewed every three days until cell passaging was performed at 80% confluence. Fluorescently labeled antibodies against NP cell markers (CD24, ab31622; KRT18, ab215839; Abcam, UK) were used to identify the phenotype of NP cells, as described previously [33]. Cells at the second passage were utilized in the subsequent experiments.

### 2.3. AOPP Preparation and Assessment

Human serum albumin (HSA) solution (30 mg/ml) was incubated with 100 mM HOCl in PBS (pH = 7.4) at ordinary temperature for 30 min. An equimolar concentration of thiosulfate was used to discontinue the reaction and block excess HOCl before the solution was dialyzed for 24 h at 4°C. AOPP-HSA were assessed according to previous description [34]. In short, the samples and 160 *μ*L citric acid (0.20 mol/L) were added into a 96-well microplate. Then, the calibrated chloramine-T model compound, 10 ml potassium iodide (KI, 1.16 mol/L in PBS), and 10 *μ*L citric acid (0.20 mol/L) were successively pipetted into the plate. Next, the absorbance was read on a spectrophotometer (Waltham, MA, USA) at 340 nm. The level of AOPP was expressed as *μ*Mol/ml of chloramine-T equivalents.

### 2.4. Protocols of NP Cell Culture and Treatment

To evaluate the impact of AOPP, the NP cells were either cotreated with incremental concentrations of AOPP (0, 100, 200, and 400 *μ*g/ml) for 24 h, or with 400 *μ*g/ml AOPP for different time points (0, 2, 6, 12, and 24 h). Allicin (purity > 98%) was bought from MedChemExpress (Shanghai, China). The cells were cotreated with incremental levels of allicin (0, 5, 10, 20, and 40 *μ*M) for 24 h to explore the effect of allicin on them. Then, to evaluate the possible protective role of allicin against AOPP-induced unfavorable influences including restrained viability and proliferation, apoptosis, oxidative stress, and mitochondrial dysfunction, the cells were pretreated with incremental concentrations (0, 5, 10, and 20 *μ*M) of allicin for 2 h and then with AOPP (400 *μ*g/ml) for 24 h. To examine whether MAPK pathways were activated in AOPP-induced oxidative damage, NP cells were treated with 400 *μ*g/ml AOPP. To explore the signaling pathway through which allicin mitigated AOPP-induced oxidative stress and apoptosis of NP cells, the cells in the experimental groups were pretreated with different concentrations of allicin (0, 5, 10, and 20 *μ*M) for 2 h, and then cotreated with AOPP (400 *μ*g/ml) for 24 h. To validate the MAPK pathway underlying the protective effects of allicin against AOPP, the NP cells were treated, respectively, with allicin (10 *μ*M), p38-MAPK inhibitor SB202190 (10 *μ*M), JNK inhibitor SP600125 (10 *μ*M), and ERK inhibitor SCH772984 (10 *μ*M) for 2 h, and then with AOPP (400 *μ*g/ml) for 24 h. To examine whether the p38-MAPK agonist can block the positive effects of allicin, the human NP cells were pretreated with allicin (10 *μ*M) or allicin (10 *μ*M) in combination with p38-MAPK activator Dehydrocorydaline chloride (Dc, 500 nM) for 2 h, then treated with AOPP (400 *μ*g/ml) for 24 h.

### 2.5. Appraisals of NP Cell Viability and Proliferation

The viability of human NP cells treated with allicin or AOPP was evaluated employing CCK-8 assay (Dojindo, Japan). Briefly, suspended human NP cells were pipetted into a 96-well plate for 24 h incubation, and then the cells were cotreated with incremental concentrations of allicin or AOPP for different time periods. Afterward, NP cells in each well were appended with 10 *μ*L solution of CCK-8 and then were cultured in the fresh medium at 37°C for 4 h. Eventually, the absorbance at 450 nm was gauged employing a spectrophotometer (BioTek, Winooski, USA).

The evaluation of NP cell proliferation was performed with a BeyoClickEdU-488 Cell Proliferation kit (Beyotime, Shanghai, China). The procedures were mainly in accord with instructions of the manufacturer. The images were acquired employing fluorescent microscopy (Olympus IX71, Japan).

### 2.6. Flow Cytometry for Analyzing Apoptosis, ROS, and MTP

Human NP cells were harvested after being treated in each group. An Annexin V-FITC Apoptosis Detection Kit (KeyGEN, China) was utilized to estimate the apoptotic levels of NP cells. The alterations of MTP were measured utilizing the cationic fluorescent indicator JC-1 (Beyotime). The ROS levels of cells were detected using the fluorescent dye dihydroethidium (DHE, Beyotime). The fluorescence intensity emitted by DHE was measured by flow cytometry, and ROS levels were expressed as mean fluorescence intensity for comparison. The final ROS level of each experimental group was expressed as a ratio relative to that of control group. All the above procedures were in accordance with previous descriptions [35]. Analysis of the sample was by dint of a FACSCalibur flow cytometer (BD Biosciences).

### 2.7. Lipid Peroxidation Detection

A malondialdehyde (MDA) Assay Kit (Beyotime, China) was used to detect the cellular lipid peroxidation. MDA is considered as a distinctive peroxidative product of lipid. Briefly speaking, NP cells were dissolved in MDA standards before blending with the working solution of thiobarbituric acid (TBA), in accordance with instructions. The absorbance of the MDA-TBA compound was gauged at 532 nm by using an automatic microplate reader (Multiskan MK3, Thermo Scientific, USA). The level of cellular MDA was reckoned on the basis of the standard curve.

### 2.8. Immunofluorescence Staining

Immunofluorescence staining was done to NP cells according to previous descriptions [36]. Primary antibodies aiming at cleaved caspase-3 (1: 400; #9579, Cell Signaling Technology) were first used to incubate with NP cells at 4°C for all night. In the second day, a continued incubation with the secondary antibodies Cy3 conjugated goat anti-rabbit IgG (1 : 200; BA1032, Boster Biological Technology) lasted for 2 h. DAPI (Beyotime) was used to stain the nuclei. Images of fluorescence were obtained via immunofluorescent microscopy (Olympus IX71, Japan) as well as confocal laser-scanning microscopy (LSM780, ZEISS, Germany). The immunofluorescence intensity values were determined using the ImageJ image analysis software (NIH). For the stained sample from each group, the mean immunofluorescence was determined by analyzing the fluorescence intensity values of three randomly selected images under the same magnification (×400). In order to compare the fluorescence intensity between groups, all the image analyses were performed using the same protocol. The final fluorescence intensity of each experiment group was expressed as a ratio relative to that of control group.

### 2.9. Western Blotting

The total and cytoplasmic, as well as mitochondrial proteins from elaborately treated NP cells were extracted employing the commercial kits, following the merchant guide (Beyotime, China). Levels of these proteins were then gauged employing an Enhanced BCA Protein Assay Kit (Beyotime, China). 10–12% SDS-PAGE gels were used to separate these proteins, which were then transferred to polyvinylidene fluoride (PVDF) membranes (Millipore, USA). The PVDF membranes were previously blocked before an overnight incubation at 4°C with primary antibodies (1 : 500–1 : 1000) and then with horseradish peroxidase- (HRP-) conjugated secondary antibodies (1 : 2000; Abcam). Enhanced chemiluminescence reagents (Amersham, Piscataway, USA) were utilized to examine the expressed proteins. Primary antibodies against the following molecules were used: Bax (ab32503, Abcam), cytochrome-c (ab133504, Abcam), cleaved caspase-3 (#9579, Cell signaling Technology), Bcl-2 (ab196495, Abcam), VDAC1 (sc-32063, Santa Cruz Bio-technology), cleaved caspase-9 (#9505, Cell Signaling Technology), GAPDH (#5174, Cell Signaling Technology), MAPK Family Antibody Sampler Kit (#8690, #4695, and #9252; Cell Signaling Technology), and Phospho-MAPK Family Antibody Sampler Kit (#4511, #4370, and #4668; Cell Signaling Technology).

### 2.10. Enzyme-Linked Immunosorbent Assay (ELISA) for AOPP Testing

After being weighted, the NP tissues were cut into pieces, mixed with PBS solution (9 ml per gram), and fully ground. Then, the sample was centrifuged (C2500-R-230V, Labnet, USA) at 1000 × g for 20 min. The supernatant was collected and tested with ELISA Kit for AOPP (Cloud-Clone Corp, USA) according to the manual. The optical density (O.D. value) of each well was measured at 450 nm using a microplate reader (Multiskan MK3, Thermo scientific). The concentrations of AOPP were calculated from the standard curve according to the manufacturer's instructions.

### 2.11. Data Analysis

All experiments were independently replicated at least three times. The analysis of data was finished with the SPSS v.18.0 software (USA). The data from all groups were presented as means ± SD (standard deviation). Contrastive analysis of means between groups was evaluated using Student's *t*-test or one-way analysis of variance (ANOVA) with post hoc analysis using the Tukey's test. Graphic analysis of the data was conducted using GraphPad Prism 7.0 (GraphPad Software, California, USA). *p* < 0.05 was the prerequisite to conclude that differences among statistics were meaningful.

## 3. Results

### 3.1. AOPP Inhibited NP Cell Viability and Proliferation and Were Accumulated in the Degenerative IVD

**Figure 1:**
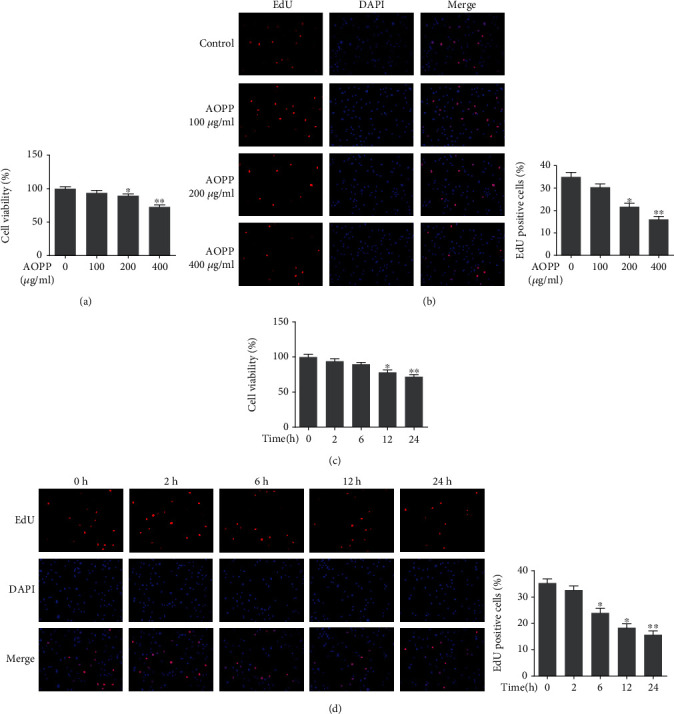
AOPP treatment inhibited human NP cell viability and proliferation in a dose- and time-dependent manner. (a) The human NP cells were treated with AOPP (0, 100, 200, and 400 *μ*g/ml) for 24 h, and 0 *μ*g/ml group served as a control. The cell viability of each group was detected by a CCK-8 assay. (b) The human NP cells were treated with AOPP (0-400 *μ*g/ml) for 24 h, and 0 *μ*g/ml group served as a control. The cell proliferation was determined using EdU staining combined with DAPI staining for the nuclei under fluorescence microscope, with the EdU positive cells quantitated. Original magnification: ×200. (c) The human NP cells were treated with 400 *μ*g/ml AOPP for 0 h, 2 h, 6 h, 12 h, and 24 h, and 0 h group served as a control. The cell viability of each group was detected by a CCK-8 assay. (d) The human NP cells were treated with 400 *μ*g/ml AOPP for 0 h, 2 h, 6 h, 12 h, and 24 h, and 0 h group served as a control. The cell proliferation was determined using EdU staining combined with DAPI staining for the nuclei under fluorescence microscope, with the EdU positive cells quantitated. Original magnification: ×200. Data were represented as mean ± SD. ^∗^*p* < 0.05 and ^∗∗^*p* < 0.01 versus the control group, *n* = 3.

To explore the impact of AOPP on the viability and proliferation of NP cells in vitro, a group of human NP cells were treated with incremental concentrations of AOPP (0, 100, 200, and 400 *μ*g/ml) made from human serum albumin (HSA) for 24 h, and then another contrastive group was treated with a high level of AOPP (400 *μ*g/ml) for different times (0, 2, 6, 12, and 24 h). Compared with the control, the AOPP-treated groups showed suppressed cell viability and proliferation, detected by CCK-8 kit and EdU staining, respectively. Notably, this visualized inhibition was both dose-dependent and time-dependent as depicted (Figures 1(a)–1(d)). Moreover, to explore the clinical relevance of the current study, we compared the levels of AOPP between the control human IVD tissues (Pfirrmann I or II) and the degenerative IVD tissues (Pfirrmann IV or V). Results showed that the levels of AOPP in degenerative tissues were significantly higher than that of control tissues (Figure S1(c)).

### 3.2. Effect of Allicin on NP Cell Viability and Proliferation

**Figure 2:**
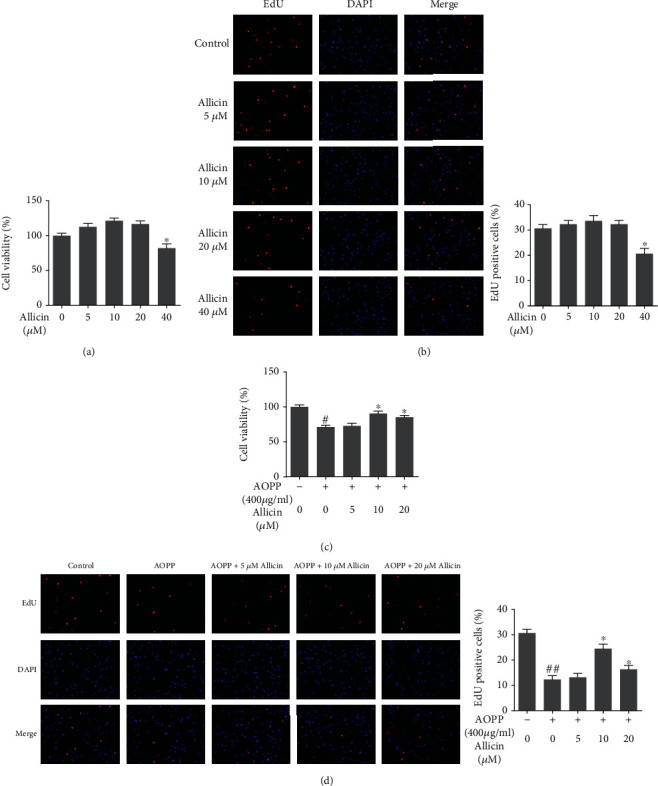
Effects of allicin on the human NP cell viability and proliferation. (a) The human NP cells were treated with allicin (0, 5, 10, 20, and 40 *μ*M) for 24 h, and 0 *μ*M group served as a control. The cell viability of each group was detected by a CCK-8 assay. ^∗^*p* < 0.05 versus the control group, *n* = 3. (b) The human NP cells were treated with allicin (0-40 *μ*M) for 24 h, and 0 *μ*M group served as a control. The cell proliferation was determined using EdU staining combined with DAPI staining for the nuclei under fluorescence microscope, with the EdU positive cells quantitated. Original magnification: ×200. ^∗^*p* < 0.05 versus the control group, *n* = 3. (c) The human NP cells pretreated by allicin (0, 5, 10, and 20 *μ*M) were treated with 400 *μ*g/ml AOPP, and the cell viability of each group was examined by the CCK-8 assay. ^#^*p* < 0.05 versus the control group, ^∗^*p* < 0.05 versus the AOPP alone treatment group, *n* = 3. (d) The cell proliferation of each group was determined using EdU staining under fluorescence microscope, with the EdU positive cells quantitated. Original magnification: ×200. Data were represented as mean ± SD. ^##^*p* < 0.01 versus the control group, ^∗^*p* < 0.05 versus the AOPP alone treatment group, *n* = 3.

To explore the cytotoxic effect of allicin on NP cells, incremental concentrations of allicin (0, 5, 10, 20, and 40 *μ*M) were separately appended to independent groups for 24 h. The CCK-8 assay and EdU staining showed that incubation with <40 *μ*M allicin enhanced the cell viability and proliferation, which reached the highest level when the allicin was around 10 *μ*M. However, the concentration growing from a critical value between 20 *μ*M and 40 *μ*M showed inhibition to cell viability and proliferation as presented in the figure (Figures 2(a) and 2(b)).

### 3.3. Allicin Improved the Viability and Proliferation of AOPP-Stimulated NP Cells

Considering the critical value of the beneficial dosage, we used 0, 5, 10, and 20 *μ*M allicin to pretreat the NP cells for 2 h, which were then cotreated with AOPP-HSA for 24 h. Unsurprisingly, allicin pretreatment attenuated the impaired viability and proliferation of NP cells under AOPP challenge, which were measured employing CCK-8 and EdU staining. In addition, the most beneficial concentration of allicin was around 10 *μ*M (Figures 2(c) and 2(d)).

### 3.4. Allicin Protected NP Cells from AOPP-Induced Apoptosis

**Figure 3:**
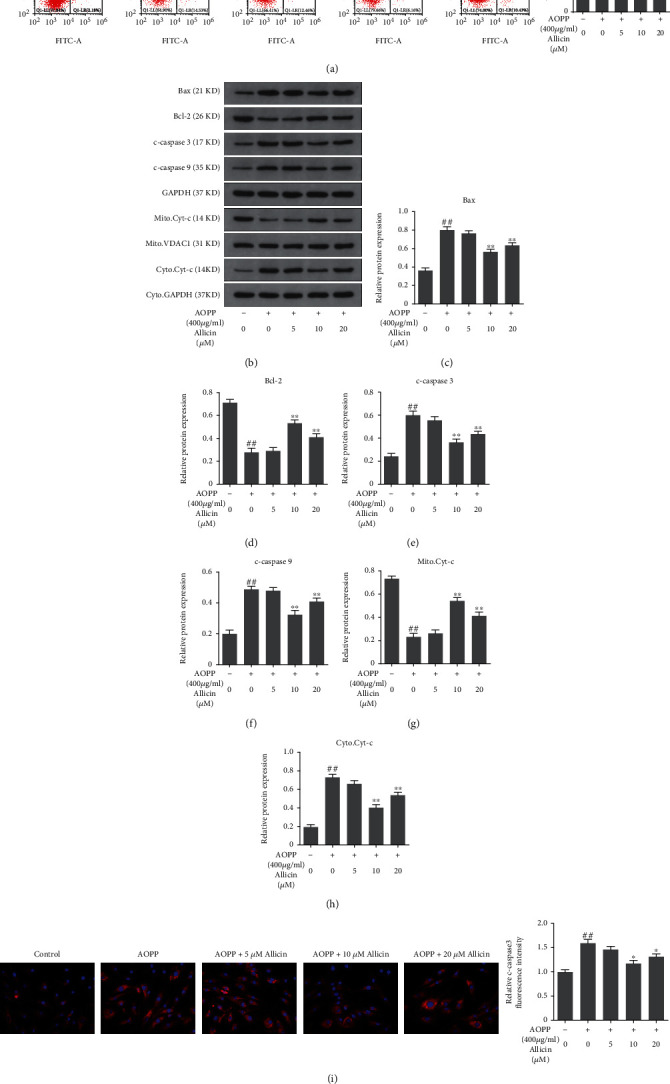
Allicin treatment alleviated AOPP-induced human NP cell apoptosis. (a) The human NP cells were pretreated with various concentrations of allicin (0, 5, 10, and 20 *μ*M) for 2 h, followed by stimulation with 400 *μ*g/ml AOPP for 24 h. And the rate of cell apoptosis was detected by flow cytometry with Annexin V-FITC/PI dual staining. The proportion of apoptotic cells in the first and fourth quadrant was measured for analysis. (b–h) The protein levels of Bax, Bcl-2, c-caspase 3, c-caspase 9, mitochondrial Cyt-c, and cytoplasmic Cyt-c were determined using Western blotting analysis (b) and quantified in (c–h). (i) Representative images of immunofluorescence staining for cleaved caspase-3 in each group, with the relative fluorescence intensity quantified. Original magnification: ×400. Data were represented as mean ± SD. ^##^*p* < 0.01 versus the control group; ^∗^*p* < 0.05 and ^∗∗^*p* < 0.01 versus the AOPP alone treatment group, *n* = 3.

Apoptosis of NP cells was detected employing the Annexin V-FITC Apoptosis Detection Kit (KeyGEN, China). After treated by 400 *μ*g/ml AOPP for 24 h, the NP cells exhibited a high apoptosis rate (>30%) compared with that (<10%) of the control group (Figure 3(a)). However, when pretreated with different levels of allicin (5, 10, and 20 *μ*M), 10 and 20 *μ*M allicin pretreatment groups showed reduced apoptosis rate compared with the AOPP alone treatment group. It was of note that 10 *μ*M seemed to be the best protective concentration of allicin against AOPP-induced apoptosis (Figure 3(a)). Also, apoptosis-associated proteins were measured by using Western blotting. As expected, common proapoptotic proteins including Bax, cleaved caspase-3, cytoplasmic cytochrome-c, and cleaved caspase-9 were highly promoted in the AOPP-treated group, while that in allicin-pretreated groups were relatively abated, especially in the 10 *μ*M allicin group. Correspondingly, antiapoptotic Bcl-2 and mitochondrial cytochrome-c were lowered in the group only treated with high levels of AOPP, while that were raised in allicin-pretreated groups, particularly in the 10 *μ*M allicin group. To detect cleaved caspase-3, another assay, immunofluorescence staining, was also employed to appraise the apoptotic differences among groups. Results showed that cleaved caspase-3 was highly expressed in AOPP group compared with allicin groups, where the level of cleaved caspase-3 dropped to varying degrees. In summary, these results indicated that allicin worked in resisting AOPP-induced apoptosis to protect NP cells.

### 3.5. Allicin Protected NP Cells from Oxidative Stress and Mitochondrial Dysfunction Induced by AOPP

**Figure 4:**
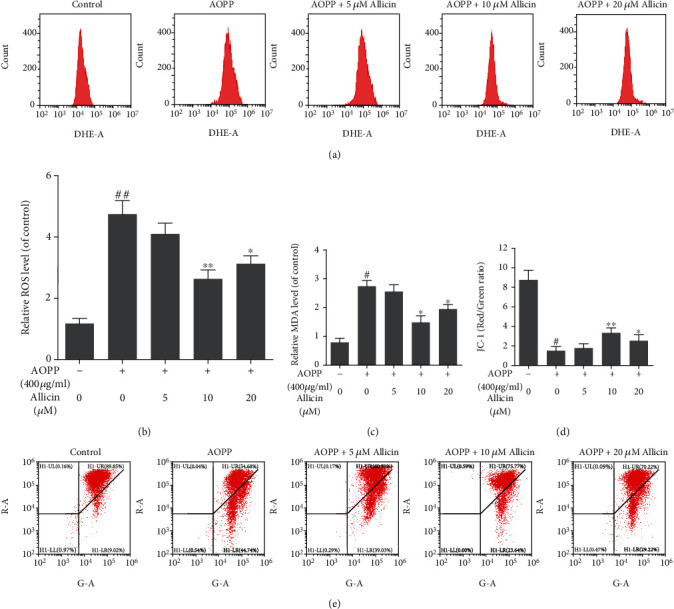
Allicin treatment inhibited AOPP-induced oxidative stress and mitochondrial dysfunction of human NP cells. (a, b) The human NP cells were pretreated with different concentrations of allicin (0, 5, 10, and 20 *μ*M) for 2 h, before treatment with 400 *μ*g/ml AOPP for 24 h. The intracellular ROS levels of the NP cells for each group were detected by ROS-specific fluorescent probe DHE and measured by subsequent flow cytometry analysis. Representative peak charts of flow cytometry and relative quantitative analysis were shown. (c) The intracellular MDA levels (as a marker of lipid peroxidation) of human NP cells were examined by a commercial kit. (d, e) The mitochondrial membrane potential of human NP cells in each group was examined by JC-1 staining and measured by subsequent flow cytometry analysis. The quantitative analysis of the ratio of red fluorescence (*y* axis) to green fluorescence (*x* axis) and representative scatter plots of flow cytometry were shown. Data were represented as mean ± SD. ^#^*p* < 0.05 and ^##^*p* < 0.01 versus the control group; ^∗^*p* < 0.05 and ^∗∗^*p* < 0.01 versus the AOPP alone treatment group, *n* = 3.

Next, we explored the mechanisms behind AOPP-induced apoptosis and how allicin antagonized it. AOPP have been reported to cause mitochondrial dysfunction through a ROS-dependent way, which might expound the apoptosis phenomenon of NP cells [37]. Here, the dihydroethidium- (DHE-) dyed products of ROS were detected by flow cytometry. The results showed that AOPP evidently increased ROS levels in NP cells, while allicin pretreatment partially restrained this effect (Figures 4(a)–4(c)). Moreover, we detected the level of MDA, a characteristic peroxidative product of lipid. We found that the AOPP challenge promoted the MDA generation, which was inhibited partially by allicin pretreatment (Figure 4(d)). To appraise the function of mitochondria under oxidative stress, we turned our attention to the MTP, whose depolarization proved the key event of intrinsic apoptosis. JC-1 assay indicated that the MTP was decreased markedly due to AOPP stimulation, while allicin pretreatment could attenuate this damage to some extent (Figure 4(e)). In summary, allicin pretreatment attenuated oxidative stress and mitochondrial dysfunction induced by AOPP in NP cells.

### 3.6. Allicin Protected against AOPP Mediated Oxidative Stress and Mitochondrial Apoptosis via Suppressing the p38-MAPK Pathway

**Figure 5:**
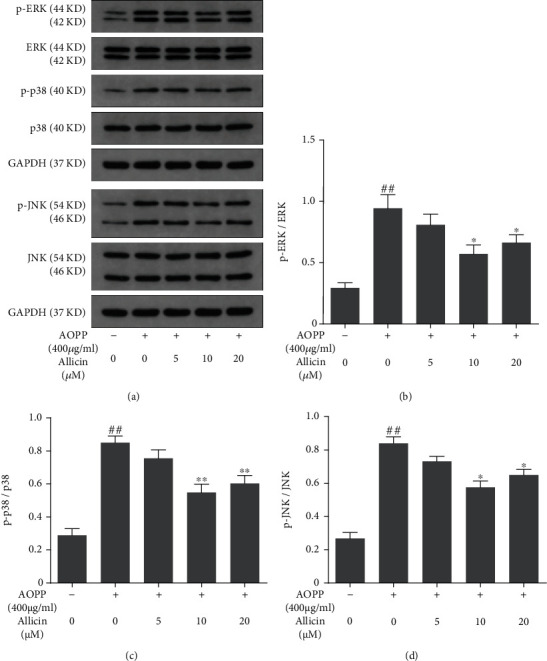
Effects of allicin on AOPP-induced MAPK pathway activation. (a) The human NP cells were pretreated with various concentrations of allicin (0, 5, 10, and 20 *μ*M) for 2 h, followed by stimulation with 400 *μ*g/ml AOPP for 24 h. The protein levels of ERK, phosphorylated ERK, p38, phosphorylated p38, JNK, and phosphorylated JNK were determined using Western blotting analysis. (b–d) Immunoblot bands corresponded to (b) p-ERK, (c) p-p38, and (d) p-JNK were quantified by densitometric analysis and normalized to their corresponding total kinase. Data were represented as mean ± SD. ^##^*p* < 0.01 versus the control group; ^∗^*p* < 0.05 and ^∗∗^*p* < 0.01 versus the AOPP alone treatment group, *n* = 3.

**Figure 6:**
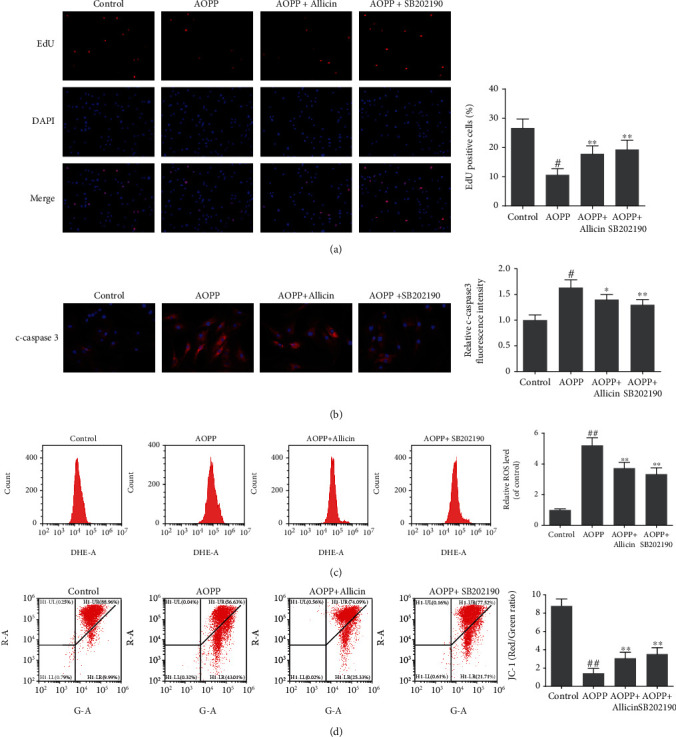
Allicin alleviated AOPP-induced oxidative stress and mitochondrial dysfunction via p38-MAPK pathway in human NP cells. (a) The human NP cells were pretreated with allicin (10 *μ*M) or p38-MAPK inhibitor SB202190 (10 *μ*M) for 2 h, then treated with AOPP (400 *μ*g/ml) for 24 h. The cell proliferation was determined using EdU staining combined with DAPI staining for the nuclei under fluorescence microscope, with the EdU positive cells quantitated. Original magnification: ×200. (b) Representative images of immunofluorescence staining for cleaved caspase-3 in each group, with the relative fluorescence intensity quantified. Original magnification: ×400. (c) The intracellular ROS levels of the NP cells for each group were detected by ROS-specific fluorescent probe DHE and measured by subsequent flow cytometry analysis. (d) The mitochondrial membrane potential of human NP cells in each group was examined by JC-1 staining and measured by subsequent flow cytometry analysis. Representative scatter plots of flow cytometry and the quantitative analysis of red fluorescence to green fluorescence ratio were shown. Data were represented as mean ± SD. ^#^*p* < 0.05 and ^##^*p* < 0.01 versus the control group; ^∗^*p* < 0.05 and ^∗∗^*p* < 0.01 versus the AOPP alone treatment group, *n* = 3.

Activation of MAPK pathways constitutes a key component in oxidative stress-associated apoptosis [38]. To ascertain the signaling pathway underlying apoptosis of NP cells stimulated by AOPP, Western blotting was performed to appraise the levels of MAPK subfamilies: p38, p-p38, ERK, p-ERK, JNK, and p-JNK. The results showed that AOPP treatment markedly increased the expression of all the above proteins associated with MAPK pathways (Figures 5(a)–5(d)). Therefore, MAPK signaling pathways might play an important role in AOPP-induced apoptosis of human NP cells. Next, we detected the levels of activated MAPK pathways in allicin-pretreated groups. Unsurprisingly, allicin pretreatment suppressed the activation of these MAPK pathways, with the best performance in 10 *μ*M allicin group. Then, the p38 inhibitor SB202190, the JNK inhibitor SP600125, and the ERK inhibitor SCH772984 were used to validate the activation of MAPK pathways. NP cells were pretreated, respectively, with allicin (10 *μ*M), SB202190 (10 *μ*M), SP600125 (10 *μ*M), and SCH772984 (10 *μ*M) for 2 h, before being treated with AOPP for 24 h. Compared with AOPP-treated group, in SB202190-treated group, we observed promoted proliferation, decreased level of ROS, downregulated cleaved caspase-3, and partial recovery of MTP, and these effects resembled the allicin-treated group (Figures 6(a)–6(d)). The inhibited expression of p38 or p-p38 in SB202190-treated group was corroborated by Western blot (Figure S1(a), (b)). However, parts of such protective effects were not found in SP600125- or SCH772984-treated groups, where the cell viability, apoptosis rate, and relative ROS levels of NP cells resembled that of the AOPP alone treatment group (Figure S2). In summary, AOPP induced oxidative stress and activated mitochondrial apoptosis in NP cells via activation of p38-MAPK pathway, while allicin could exert protective effects against AOPP by inhibition of such signaling pathway.

### 3.7. p38-MAPK Agonist Could Block the Effects of Allicin on AOPP-Induced Oxidative Stress and Mitochondrial Apoptosis

**Figure 7:**
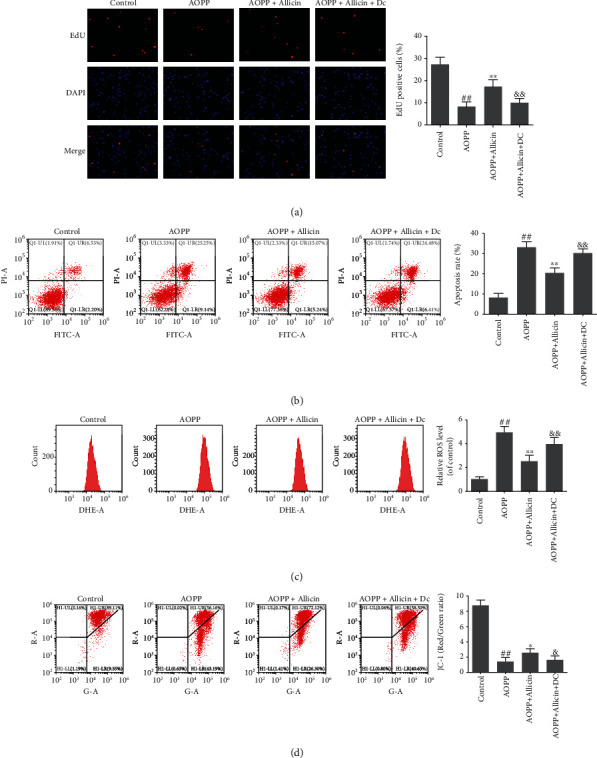
Allicin alleviated AOPP-induced oxidative stress and mitochondrial dysfunction via p38-MAPK pathway in human NP cells. (a) The human NP cells were pretreated with allicin (10 *μ*M) or allicin (10 *μ*M) in combination with p38-MAPK activator Dehydrocorydaline chloride (Dc, 500 nM) for 2 h, then treated with AOPP (400 *μ*g/ml) for 24 h. The cell proliferation was determined by EdU staining combined with DAPI staining for the nuclei under fluorescence microscope, with the EdU positive cells quantitated. Original magnification: ×200. (b) The cell apoptosis rate was detected by flow cytometry with Annexin V-FITC/PI dual staining. The proportion of apoptotic cells in the first and fourth quadrant was measured for analysis. (c) The intracellular ROS levels for each group were detected by ROS-specific fluorescent probe DHE and measured by subsequent flow cytometry analysis. (d) The mitochondrial membrane potential of human NP cells in each group was examined by JC-1 staining and measured by subsequent flow cytometry analysis. Data were represented as mean ± SD. ^##^*p* < 0.01 versus the control group, ^∗^*p* < 0.05 and ^∗∗^*p* < 0.01 versus the AOPP alone treatment group, and ^&^*p* < 0.05 and ^&&^*p* < 0.01 versus the AOPP+allicin treatment group, *n* = 3.

To further examine whether inhibited p38-MAPK pathway actually functioned in allicin-mediated protection against AOPP-induced oxidative stress and mitochondrial apoptosis, the p38-MAPK activator Dehydrocorydaline chloride (Dc) was used. The human NP cells were pretreated with allicin (10 *μ*M) or allicin (10 *μ*M) in combination with Dc (500 nM) for 2 h, and then treated with AOPP (400 *μ*g/ml) for 24 h. The restrained cell proliferation, promoted apoptosis, ROS generation, and decrease of MTP induced by AOPP were mitigated to varying degrees in allicin pretreatment group, while these protective effects of allicin were markedly blocked by p38-MAPK agonist Dc (Figure 7). In short, inhibition of the p38-MAPK pathway was an important avenue mediating the effect of allicin on AOPP-induced oxidative stress and mitochondrial apoptosis.

## 4. Discussion

LBP is a cosmopolitan problem that has become not only a medical issue, but also a socioeconomic burden in contemporary society [39]. IDD is the main contributor to LBP [40]. Although many efforts have been made to alleviate the symptoms of IDD, valid pharmacotherapy to inhibit IDD progression is still missing, possibly due to an insufficient understanding of its etiology [17, 41]. As a joint outcome of various initiators, abnormal apoptosis of NP cells has been widely detected in degenerative discs and is considered one of the most common pathogenetic mechanisms of IDD [9]. In fact, the extent of apoptosis of cells in regressive disc specimens is 53%~73%, as reported previously [42].

Apoptotic pathways include the death receptor (also called the extrinsic) pathway and the mitochondrial (also called the intrinsic) pathway [43]. Cytochrome-c release, apoptotic body formation, and activating the caspase cascade are necessary components of the mitochondrial apoptotic pathway, which can be initiated by diverse factors including intracellular oxidative stress [44]. A newly described marker of oxidative stress in the serum is represented by AOPP, a group of protein products that contain dityrosine residues and are produced by oxidants, such as hypochlorous acid and chloramines secreted by activated neutrophils [45, 46]. However, recent studies have shown that AOPP are not only biomarkers of oxidative stress, but also a new type of oxidative pathogenic mediator [17]. To date, AOPP have been reported to be involved in several diseases, including progressive nephropathies and neuroinflammation, via a redox-dependent way [47, 48]. For example, a previous study showed that extracellular accumulation of AOPP triggered the production of ROS and induced apoptosis of dorsal root ganglion neurons [49]. However, it has not yet been elucidated whether the accumulation of AOPP can lead to apoptosis of human NP cells, which plays a key role in the pathogenesis of IDD. In the present study, we found disparities of the AOPP levels in between healthy (Pfirrmann I or II) and degenerated (Pfirrmann IV or V) human NP tissues. The levels of AOPP in the degenerated specimens were much higher than that in the healthy samples, which might suggest a pathogenic function of AOPP in IDD progression.

To explore the effects of AOPP on IDD, we separated NP cells from the healthy samples for further research. For the human NP cell isolation, since excessive time of digestion may be harmful to the cell viability, we chopped the NP tissue into small pieces to digest faster [50, 51]. In addition, a noteworthy difficulty for us in primarily culturing human NP cells was contamination, and the combined use of penicillin and streptomycin effectively prevented microbial contamination in the cell culture. Next, we exposed well-grown human NP cells of second passage to different levels of AOPP and assessed cell viability, proliferation, and apoptosis using the CCK-8 assay, EDU staining, and flow cytometry, respectively. The results showed that AOPP restrained the activity and proliferation of NP cells in both concentration- and time-dependent manners and promoted cell apoptosis.

After treating NP cells with AOPP, we detected the overexpression of proapoptotic proteins, including Bax, cytochrome-c, cleaved caspase-9, and cleaved caspase-3, while the level of the antiapoptotic protein, Bcl-2, was downregulated. This might be related to impaired mitochondrial function. Moreover, previous research found that AOPP could augment the level of ROS by activating NADPH oxidase, thus inducing preosteoblast apoptosis [19]. A leading source of cellular oxidative stress, ROS, is mainly derived from mitochondria, being generated in the electron transport chain [52, 53]. Previous studies indicated that excess ROS increased the Bax/Bcl-2 ratio and damaged key intracellular targets, such as the mitochondrial membrane, thereby promoting apoptosis through the mitochondrial apoptotic pathway [52, 54, 55]. In the prophase of mitochondrial apoptosis, the mitochondrial membrane is oxidatively damaged after attack by ROS, resulting in enhanced mitochondrial outer membrane permeability and a decreased MTP [56–58]. Moreover, ROS overproduction also increases Bax/Bcl-2 ratio, which can further impair the mitochondrial membrane and decrease the MTP [55]. Depolarization of the MTP, as a characteristic change in the forepart of apoptosis, contributes to the release of apoptosis-inducing factor and cytochrome-c from mitochondria, which in turn triggers the activation of caspase-9 and caspase-3, and the subsequent cascade of caspases, leading to the irreversible process of apoptosis [57, 59–62]. Therefore, our results indicated that AOPP might trigger the mitochondrial apoptosis pathway.

To further explore the mechanism underlying apoptosis, we first measured the intracellular levels of two main indicators of oxidative stress after treatment of NP cells with AOPP: ROS and malondialdehyde (MDA), a specific marker for lipid oxidation. As expected, the levels of both compounds were elevated, demonstrating the existence of AOPP-induced oxidative stress in NP cells. Then, we measured the MTP using the cationic fluorescent indicator, JC-1. The results showed depolarization of the MTP, suggesting mitochondrial dysfunction. A recent study targeting human chondrocytes revealed that AOPP could cause mitochondrial dysfunction in a ROS-dependent manner and induce apoptosis, in line with our findings [37].

As a natural compound extracted from garlic bulbs, allicin has been shown to exert potent antioxidant properties and is thus used as an antioxidant agent for the treatment of many diseases [63]. Previous studies have shown that allicin can reduce ROS levels, abate lipid peroxidation, and protect cells from apoptosis [28, 30, 64, 65]. Considering the prominent antioxidant properties of allicin, we conjectured that it could antagonize AOPP-induced oxidative injury to NP cells and inhibit apoptosis. Therefore, we used AOPP as an oxidative source and explored the function of allicin. NP cells were pretreated with different levels of allicin before exposure to high concentrations of AOPP (400 *μ*g/ml). Compared with the AOPP alone treatment group, allicin-pretreated NP cells exhibited less apoptosis and a lower Bax/Bcl-2 ratio, as well as reduced expression of apoptosis-related proteins including cleaved caspase-3 and -9 and cytochrome-c, demonstrating that allicin could mitigate AOPP-induced apoptosis of NP cells.

We also measured the intracellular levels of ROS and MDA, which were decreased in allicin-pretreated groups compared with the AOPP alone treatment group. This supported the antioxidative capability of allicin. In addition, by measuring MTP, we showed that AOPP-induced mitochondrial damage in NP cells was attenuated in the allicin-pretreated groups. Overall, our results showed that allicin could mitigate AOPP-induced oxidative stress and activation of the mitochondrial apoptosis pathway through its antioxidative properties in human NP cells, consistent with the results of Hong et al. [66] who discovered that allicin abated acrylamide-induced oxidative stress in BRL-3A cells. However, it is possible that these results do not represent the intracorporal effect of allicin, whose elucidation requires further experiments.

Mitogen-activated protein kinase (MAPK) signaling pathways are important avenues for apoptosis. MAPK is comprised of three chief subgroups: p38-MAPK, extracellular signal-regulated kinase (ERK), and c-Jun N-terminal kinase (JNK) [67]. Previous studies have shown that the MAPK pathway is significantly involved in cell proliferation, differentiation, and apoptosis and can be activated by various inflammatory cytokines and cellular stressors, such as ultraviolet rays, DNA damage, and oxidative stress [68, 69]. In particular, ROS engendered by NADPH oxidase are potent MAPK activators [70, 71]. Therefore, we choose to assess these primary members of the MAPK family to ascertain the signaling pathway underlying AOPP-induced apoptosis in NP cells and the protective role of allicin. The NP cells were treated with high levels of AOPP (400 *μ*g/ml) before determining the levels of p38, ERK, JNK, and their phosphorylation forms. All three MAPK subfamilies were significantly activated by AOPP administration. According to previous studies, it is of note that p38-MAPK signaling pathway could also be activated in a time-dependent manner by various factors, such as LPS, TNF-alpha, and IL-1beta [72, 73]. A similar assay was performed following treatment with different concentrations of allicin for 2 h, and then with the highest level of AOPP (400 *μ*g/ml) for 24 h. Notably, we observed concentration-dependent inhibition of all the three MAPK pathways in the allicin-pretreated groups. Afterwards, p38 inhibitor SB202190, JNK inhibitor SP600125, and ERK inhibitor SCH772984 were used to validate the pathway involved in AOPP-induced apoptosis of NP cells and in the antagonistic effect of allicin against AOPP. It was of note that though all MAPK subfamilies were activated, the protective function against AOPP was found only when the p38, but not the JNK or ERK, was inhibited. The extent of apoptosis and the levels of ROS of the SB202190-treated group were significantly lower than that of the AOPP alone treatment group and were similar with that of allicin-treated group. Correspondingly, our results of Western blotting showed that the expression of p38 or p-p38 was decreased by allicin pretreatment. Next, we used the p38-MAPK activator Dc to further validate the role of inhibited p38-MAPK pathway in allicin-mediated protection against AOPP-induced oxidative stress and mitochondrial apoptosis. In the case of levels of apoptosis, MTP, ROS, and cell proliferation, the allicin and Dc combined pretreatment group was much closer to AOPP alone treatment group than the allicin alone pretreatment group, suggesting that the anti-AOPP effects of allicin were blocked by Dc. In summary, our experimental results revealed that allicin could mitigate AOPP-induced oxidative stress and mitochondrial apoptosis in NP cells by inhibiting activation of the p38-MAPK pathway.

In conclusion, our findings provide new insights into the etiology of apoptosis in NP cells and, more importantly, suggest that allicin may be a novel remedy to mitigate the progression of IDD. However, there are a few limitations to our study. Because all our observations were based on in vitro experiments, further research is required to validate these findings in vivo. In addition, because the use of allicin as a multifunctional medicine has a long history, its protective role in NP cells may also involve other mechanisms. Indeed, a prior study showed that allicin could mitigate the dexamethasone-induced abnormal expression of cytochrome-c, c-caspase 3, c-caspase 9, Bax, and Bcl-2 through activating the PI3K/AKT pathway, and thus rescued apoptosis of osteoblasts induced by dexamethasone in rats [74]. Another study revealed that allicin attenuated age-related cognitive dysfunction through activating Nrf2 antioxidant signaling pathways [75]. Therefore, further investigations focusing on molecular mechanisms are required to shed light on the antiapoptotic function of allicin against AOPP in NP cells.

Recently, the comparability between 3D culture in hypoxia and IVD microenvironment aroused our interest. The NP cells mainly obtain oxygen and nutrients through the osmosis of the cartilage endplate (CEP). During IDD, unexplained calcification of the CEP usually blocks this effect and helps to form hypoxic areas. Therefore, NP cells in IDD are likely to be hypoxic. In a 2D monolayer, all cells are exposed to the gas phase, but the internal area of the 3D culture is usually limited by diffusion, leading to hypoxia and necrosis [76]. Similarly, the distribution of NP cells is also 3D due to the thickness of IVD, which means that the internal cells are more prone to be anoxic than the cells close to the CEP. Therefore, 3D culture in hypoxia can better mimic the microenvironment in native IVDs. In addition, studies have shown that hypoxia can also influence intracellular oxidative stress. For example, exposure to hypoxia could restrict the generation of ROS in NP cells and inhibit apoptosis [77–79]. It can be inferred that hypoxia, a nonignorable variable in the human IVD, might have an impact on our research and change the existing experimental results of AOPP. Hence, applying 3D culture in hypoxia might better reflect the actual level of oxidative stress and apoptosis induced by AOPP in human NP cells. It is also helpful to better determine the pharmacodynamic curve of allicin for that the antioxidative function of allicin might be attenuated by hypoxia. Therefore, we are very interested in 3D culture under hypoxic conditions and hope to determine the effects of AOPP on NP cells in this context, which inspired our follow-up research. At the same time, more investigations in the future are needed to uncover the role of oxidative stress in IDD by using 3D culture in hypoxia.

## Data Availability

The data used to support the findings of this study were included within the article.

## Abbreviations

AOPP: Advanced oxidation protein products
LBP: Low back pain
IVD: Intervertebral disc
IDD: Intervertebral disc degeneration
NP: Nucleus pulposus
ECM: Extracellular matrix
AF: Annulus fibrosus
MAPK: Mitogen-activated protein kinase
ERK: Extracellular signal-regulated kinase
JNK: c-Jun N-terminal kinase
ROS: Reactive oxygen species
DHE: Dihydroethidium
MDA: Malondialdehyde
MTP: Mitochondrial transmembrane potential.

## Supplementary Materials

Supplementary Materials Supplementary Figure S1: allicin alleviated AOPP-induced oxidative stress and mitochondrial dysfunction via p38-MAPK pathway. Supplementary Figure S2: allicin alleviated AOPP-induced oxidative stress and mitochondrial dysfunction via p38-MAPK pathway. Supplementary Figure S3: the schematic diagram illustrating that allicin attenuated AOPP-induced oxidative stress and mitochondrial apoptosis in human NP cells.
