# Supplementary material for: Allicin Attenuated Advanced Oxidation Protein Product-Induced Oxidative Stress and Mitochondrial Apoptosis in Human Nucleus Pulposus Cells
Source: Oxid Med Cell Longev. 2020 Dec 14;2020:6685043. doi: 10.1155/2020/6685043 (PMC7758128; doi:10.1155/2020/6685043)
Supplement: Supplementary Materials — Supplementary Figure S1: allicin alleviated AOPP-induced oxidative stress and mitochondrial dysfunction via p38-MAPK pathway. Supplementary Figure S2: allicin alleviated AOPP-induced oxidative stress and mitochondrial dysfunction via p38-MAPK pathway. Supplementary Figure S3: the schematic diagram illustrating that allicin attenuated AOPP-induced oxidative stress and mitochondrial apoptosis in human NP cells. [file 6685043.f1.pdf]

Supplementary Figures

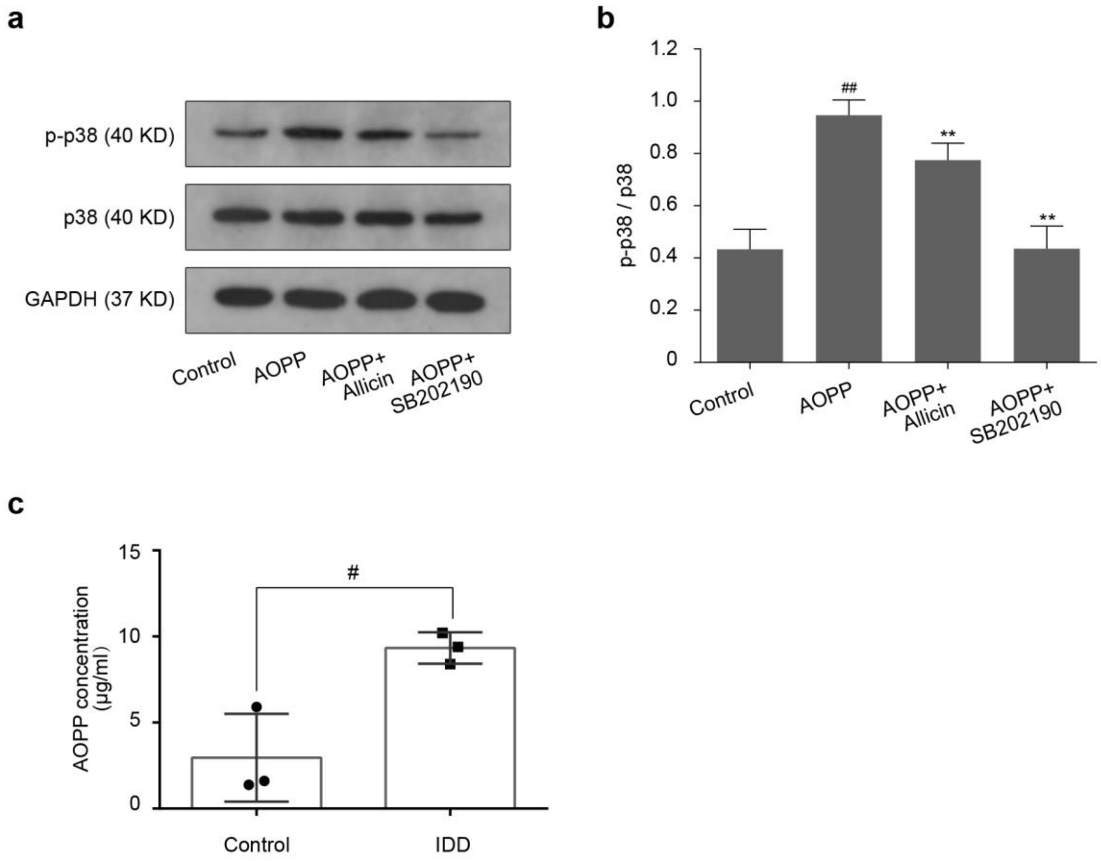

Supplementary Figure S1. Allicin alleviated AOPP-induced oxidative stress and mitochondrial dysfunction via p38-MAPK pathway. (a) The human NP cells were pre-treated with Allicin (10 μM) or p38 MAPK inhibitor SB202190 (10 μM) for 2 h, then treated with AOPP (400 μg/ml) for 24 h. The protein levels of p38, phosphorylated p38 were determined using western blotting analysis. (b) Immunoblot bands were quantified by densitometric analysis and p-p38 level was normalized to total p38 level. Data were represented as mean ± SD. $^{\#\#}p < 0.01$ versus the control group, $^{**}p < 0.01$ versus AOPP alone treatment group, n=3. (c) Relative AOPP levels of human NP tissue sample from control (n=3) and IDD (n=3) group were determined by using a human AOPP ELISA test kit. Specially, 1 gram of tissue sample was added to 9 milliliter of PBS to grind and mix, then the supernatant after centrifuging was used as the tested sample. Data were represented as mean ± SD. $^{\#}p < 0.05$ versus the control group.

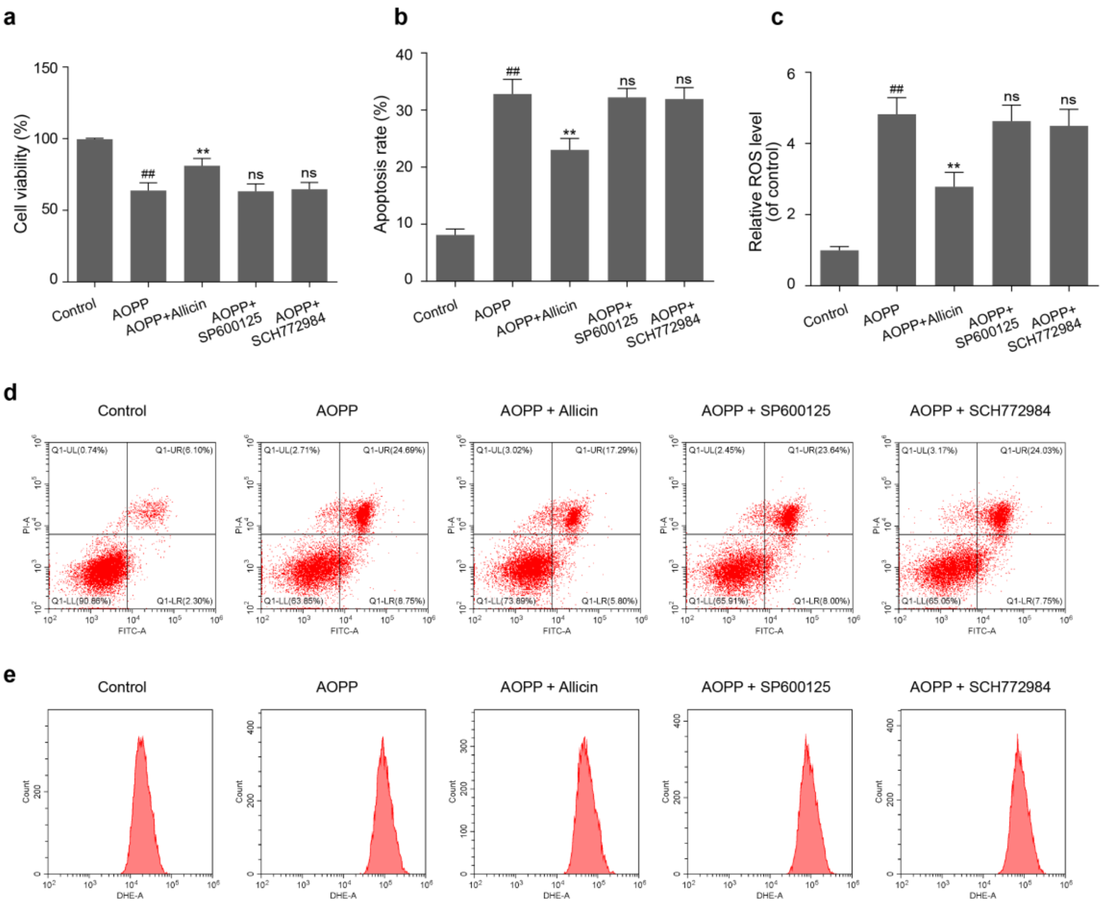

Supplementary Figure S2. Allicin alleviated AOPP-induced oxidative stress and mitochondrial dysfunction via p38-MAPK pathway. (a) The human NP cells were pre-treated with Allicin (10 μM) or JNK inhibitor SP600125 (10 μM) or ERK inhibitor SCH772984 (10 μM) for 2 h, then treated with AOPP (400 μg/ml) for 24 h. The cell viability of each group was detected by a CCK-8 assay. (b, d) The rate of cell apoptosis was detected by flow cytometry with Annexin V-FITC/PI dual staining. The proportion of apoptotic cells in the first and fourth quadrant was measured for analysis. (c, e) The intracellular ROS levels of the NP cells for each group were detected by ROS-specific fluorescent probe DHE and measured by subsequent flow cytometry analysis. $^{\#\#}p < 0.01$ versus the control group, $^{**}p < 0.01$ versus AOPP alone treatment group, ns = no significance compared with AOPP alone treatment group, n=3.

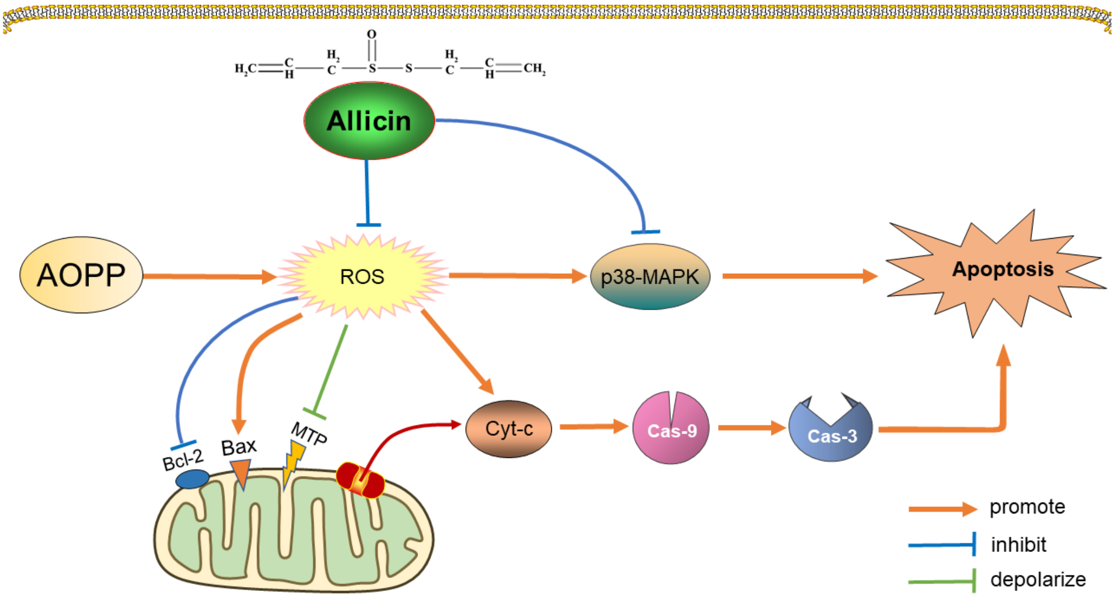

Supplementary Figure S3. The schematic diagram illustrating that allicin attenuated AOPP-induced oxidative stress and mitochondrial apoptosis in human NP cells.
